# Robotic Repair of a Congenital Isolated Cleft of Anterior Tricuspid Valve Leaflet

**DOI:** 10.1177/2324709618823809

**Published:** 2019-01-15

**Authors:** Mohanad Hamandi, Karim Al-Azizi, Alexander Crawford, Joy Fan, J. Michael DiMaio, Robert L. Smith

**Affiliations:** 1 Baylor Scott& White Research Institute at the Heart Hospital, Plano, TX, USA

**Keywords:** tricuspid cleft, robotic repair, congenital valve disease

## Abstract

Congenital isolated tricuspid valve (TV) cleft in the anterior leaflet is a rare occurrence, while clefts of the mitral valve leaflets are more common and are usually associated with other congenital heart diseases. In this article, we report a case of TV regurgitation in a young adult female due to an isolated congenital cleft in the anterior TV leaflet, which was surgically repaired using a minimally invasive robotic approach.

## Introduction

Severe tricuspid valve (TV) regurgitation due to a cleft in the anterior leaflet is a rare congenital anomaly.^1^ Although the pathogenesis is still unknown, an abnormality in the development of the endocardial cushion is suspected.^2^ This lesion can often be seen with atrial septal defects, perimembranous ventricular septal defects, or pulmonary valve stenosis.^1,3^ We report a case of isolated congenital tricuspid regurgitation caused by a cleft in the anterior tricuspid leaflet in an adult and correction via a robotically assisted minimally invasive approach.

## Clinical Summary

A 39-year-old woman was referred to our multispecialty valve clinic with progressively worsening dyspnea on exertion. For the past several years, the patient has had mild dyspnea on exertion with significant worsening over the past 6 months. She was in New York Heart Association functional class III. Physical findings and electrocardiography included 3/6 systolic murmur and regular sinus rhythm, respectively. Transthoracic echocardiography revealed a markedly dilated right atrium and right ventricle with severe tricuspid annular dilatation and regurgitation (Video 1 available online). The patient was evaluated by the heart team and deemed an appropriate candidate for robotically assisted TV surgery.

**Figure 1.**
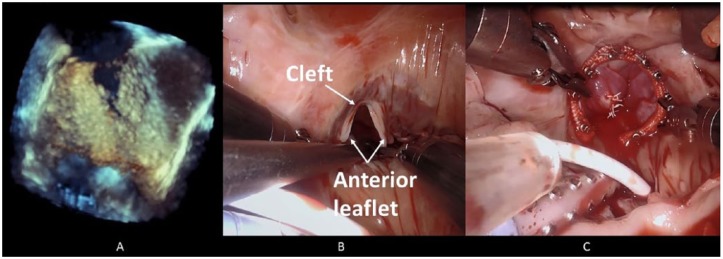
(A) Intraoperative transesophageal echocardiography shows a cleft in the anterior TV leaflet. (B and C) Intraoperative robotic images show a large cleft in the anterior TV leaflet before and after suture repair with tricuspid annuloplasty.

Preoperative assessment showed that the patient was an appropriate candidate for femoral cannulation. The aortic balloon endoclamp was placed via the arterial cannula. Robotic ports were placed in a standard fashion for mitral valve surgery. The heart was arrested with antegrade and retrograde cold del Nido cardioplegia. After right atriotomy, an isolated cleft in the anterior leaflet of the TV extending less than 0.5 cm to the annulus was found (Figure 1B). There was no sign of trauma nor evidence of structural valve disease in the subvalvular apparatus. The free edges of the anterior leaflet were carefully approximated using 6-0 Gore-Tex suture. It had no chordal support and had rolled edges indicative of a primary congenital etiology. It also showed marked annular dilation; therefore, a 25 Simulus ATS annuloplasty band was secured from the midseptal annulus around the anteroseptal commissure (Figure 1C). The valve had excellent competency when tested with saline.

The patient had a body mass index of 35.2 kg/m^2^, yet was discharged home on postoperative day 5 after an uneventful hospital course. She was able to resume driving 1 week after discharge and reported significant improvement in her functional status and better exercise tolerance at 30-day and 1-year follow-up. Echocardiographic assessments demonstrated remodeling of the right atrium and ventricle to normal size and right ventricular systolic function. Transthoracic echocardiography also showed no regurgitant TV and normal leaflet motion (Video 2; available online).

## Discussion

TV clefts have been previously reported but were associated with other pathologies including atrial septal defect, perimembranous ventricular septal defect, or pulmonary valve stenosis.^1,3^ The pathology seen in this case report is remarkable in that it was tricuspid regurgitation caused by an isolated anterior leaflet cleft. Congenital clefts of the mitral valve are much more common and have been associated with atrioventricular canal defects.^1^ The etiology of this tricuspid anomaly is unknown but has been hypothesized to be due to a developmental defect of the endocardial cushion.^1^ Tricuspid clefts have been corrected via an open surgical procedure in which the cleft is repaired and tricuspid annuloplasty is performed.^1-3^ We report the first use of a robotically assisted minimally invasive approach to repair this tricuspid pathology.

It is important to recognize that a tricuspid congenital defect can cause heart failure and right heart dysfunction. At the 1-year follow-up, this patient presented with no signs of heart failure and was in New York Heart Association Class I with right atrial and ventricular remodeling. With the advancement of imaging modalities, diagnosis of unusual conditions such as this are more likely. As we have shown, the advancement of technology has also allowed for a safe repair of this defect by a minimally invasive robotic approach.
